# Early Detection and Surveillance of Infectious Disease Outbreaks in Nigeria: Integrating Nigerian Pidgin English (NPE) into the EPIWATCH^®^ Platform

**DOI:** 10.1007/s10916-026-02340-1

**Published:** 2026-02-13

**Authors:** Omolara Kolawole, Ashley Quigley, Abrar A. Chughtai, Chandini R. MacIntyre

**Affiliations:** 1 Biosecurity Program, The Kirby Institute, Kensington, NSW Australia; 2 School of Population Health, University of New South Wales, Sydney, Australia

**Keywords:** Infectious disease surveillance, Artificial intelligence, Natural language processing, Nigerian Pidgin English, Open-source intelligence (OSINT), Early outbreak detection

## Abstract

This study aimed to integrate Nigerian Pidgin English (NPE) into the Artificial Intelligence system EPIWATCH^®^, with the aim of providing early detection and enhanced surveillance of infectious disease outbreaks in Nigeria. The widespread use of NPE as a lingua franca, spoken by over 75 million people in Africa, improves interaction and communication within communities across Nigeria. Key search terms were translated into NPE and incorporated into EPIWATCH^®^ for surveillance. Using publicly available outbreak reports collected by EPIWATCH^®^ from Nigeria between 2018 and 2023, we conducted a descriptive analysis to compare outbreak detection patterns before and after the integration of NPE into the system. We observed a 315% increase in EPIWATCH^®^ outbreak reports in March 2024 compared to the March median across 2018 to 2023, following NPE language integration. This study demonstrates the potential of leveraging technology and linguistic diversity to improve disease surveillance using open source intelligence (OSINT) and response efforts in Nigeria. Findings should be interpreted in light of the short prospective time frame, scope, contextual factors, and public source constraints, which may limit generalisability and nuance in assessing the impact of NPE integration.

**Supplementary Information:**

The online version contains supplementary material available at 10.1007/s10916-026-02340-1.

## Introduction

In recent years, the global health community has gradually recognized the importance of the early detection of disease outbreaks together with a coordinated, public health-focused response [1, 2]. Nigeria, with a population exceeding 200 million, struggles significantly with infectious diseases [3]. The country has one of the highest Tuberculosis burdens globally, whilst malaria is a major cause of illness and mortality, particularly for pregnant women and children under five. There have also been several significant infectious disease outbreaks in recent years such as the meningitis outbreak that occurred in 2017, primarily caused by *Neisseria meningitidis* serogroup C, which affected over 14,000 people, and resulted in more than 1,100 deaths [4, 5]. Similarly, in 2018, there was a resurgence of Lassa fever, a viral haemorrhagic fever transmitted by rodents, with over 1,000 open source outbreak reports and a high case fatality rate [6].

One of the main challenges with disease response in Nigeria is the healthcare system’s limited ability to quickly identify, diagnose, and contain outbreaks [7]. This is reflected in long wait times for medical care due to inadequate healthcare infrastructure, particularly in rural areas. Vaccine hesitancy and widespread misinformation further complicate disease control efforts, especially during immunisation campaigns. In addition, gaps in disease surveillance and reporting hinder the timely identification and containment of outbreaks [8].

Despite this, Nigeria has overcome many obstacles to improve laboratory diagnostics, bolster disease surveillance, and launch vaccination campaigns, all of which have increased the country’s capacity to respond to diseases [9, 10]. This improvement is because of cooperation between foreign partners, healthcare professionals, and the government.

In the late 20th and early 21 st centuries, public health surveillance experienced significant advancements including the use of electronic health records, syndromic surveillance, big data analytics, mobile health technologies, and genomic surveillance, partly due to increased global travel and trade [11, 12]. Open source intelligence (OSINT) has played a crucial role in this progress, providing real-time data and insights into disease outbreaks from sources like the internet and social media. The use of OSINT is valuable for disease surveillance and early response efforts, enabling authorities to promptly and efficiently identify and address outbreaks in circumstances with limited resources such as when healthcare facilities are overloaded, as was the case during the COVID-19 pandemic [9, 12, 13]. Historically, manual reporting from medical facilities, community health workers, and laboratories has been a mainstay of grassroots disease surveillance methods. With the use of paper-based forms or phone calls to health authorities, data on disease cases, symptoms, and trends are gathered. Traditional surveillance can therefore be slow and may miss real-time data, even though they are valuable.

Conversely, surveillance systems with Artificial Intelligence (AI) [14], such as EPIWATCH^®^, can automatically analyse massive volumes of data from multiple sources. EPIWATCH^®^, which was launched in 2016, is an open source surveillance system monitoring epidemics by analysing social media and news for early signs of infectious diseases before official reports. The platform uses AI algorithms to scan social media posts to identify disease trends or symptoms and provide early warnings for outbreaks. By integrating AI technologies with traditional surveillance methods, EPIWATCH^®^ enhances disease surveillance locally, leading to improved early detection, prediction, and outbreak response [15]. Retrospectively, EPIWATCH^®^ was able to detect early outbreak signals for the West African Ebola outbreak [16], the Zika virus outbreak [17], and monitor polio eradication efforts [18], demonstrating how crucial monitoring is for quickly recognizing and addressing threats to the public’s health [14].

Currently, health information in Nigeria is disseminated through traditional media like radio and television, as well as digital channels including the NPE service of the BBC [19, 20]. Public health officials, healthcare facilities, and community health workers also play key roles in educating the public about health issues and promoting healthy behaviours [3]. However, Nigeria’s diverse linguistic landscape complicates the dissemination of health information during outbreaks due to communication barriers, resource limitations, cultural considerations, and fragmented and inconsistent health messaging that leads to confusion among the population [21, 22]. Given that rapid understanding and adherence to control measures are paramount for an effective response to outbreaks [13], language barriers can impede effective communication and coordination during public health emergencies [23, 24].

Whilst EPIWATCH^®^ searches 45 languages for outbreak news and significant health events as of 2023, it has included only 3 languages used for monitoring outbreak information pertaining to Nigeria, namely English (7%), French (1%), and Arabic (1%); though French and Arabic are not indigenous to the country) [22, 25, 26]. This not only demonstrates EPIWATCH^®^’s usefulness for surveillance but also highlights its possibly limited reach in Nigeria because of language barriers [25]. To address this, NPE, also known as “broken English”, is proposed as a solution due to its widespread use in Nigeria by over 75 million people, inclusivity, and cross-cultural applicability [3]. The versatility of NPE makes it more inclusive in this surveillance context than focusing on any single indigenous language, which justifies its selection for EPIWATCH^®^ language integration, for enhanced surveillance in Nigeria [27, 28]. By integrating NPE into EPIWATCH^®^, this project aims to overcome language barriers and create a more inclusive and responsive OSINT-based outbreak surveillance system in Nigeria [29]. Incorporating widely spoken local language search terms is intended to improve the detection and reporting of infectious disease events.

The study focuses on demonstrating the value of using NPE for OSINT surveillance by analysing outbreak data, enhancing linguistic accessibility, developing an integration framework, and assessing the impact of language-based interventions on public health outcomes.

The main objectives of this study were [1] to conduct a descriptive analysis of infectious diseases in Nigeria over a five-year period, and [2] to integrate NPE into EPIWATCH^®^ to strengthen infectious disease surveillance in Nigeria.

## Methods

### Study Design and Settings

The study was conducted in two phases over two study periods to allow for retrospective and prospective data review. Initially, an analysis of the frequency of outbreaks and disease trends in Nigeria over the past five years (2018–2023) was performed from data extracted from EPIWATCH^®^. Subsequently, an evaluation of the efficacy of existing EPIWATCH^®^ key terms in the NPE language for surveillance in Nigeria was conducted over a four-week period. EPIWATCH^®^ is intended to track and monitor disease outbreaks through data collection from multiple sources, identify outbreaks, alert generation, response effort coordination, and monitoring and assessment of interventions’ efficacy [30–32].

In the first part of this study, a descriptive analysis of outbreaks in Nigeria over a five-year period from January 1, 2018, to December 31, 2023, was conducted using EPIWATCH^®^ data. The data included monthly reports of outbreaks across the country’s 36 states, whilst the analysis focused on variables such as outbreak frequency and geographic distribution. Ethics approval was not required as all data was open sourced. The monthly data analysis allowed for trend analysis and comparison with new data generated in the first part of the study.

Descriptive statistics of the cleaned dataset was used to describe the outbreak trends of surveillance data from EPIWATCH^®^ records of all states in Nigeria over a five-year period (2018–2023). Data cleaning included resolving missing data, removing duplicates, standardizing data formats, correcting typos, errors, or outliers that could skew the analysis, converting dates to a standard format, converting categorical data to a consistent one format, ensuring data consistency, and converting data into an appropriate format and validating data against predefined rules. Relevant data on the number of reported outbreaks and their location was retrieved, sorted, and examined for completeness.

For the second part of the study, a list of key search terms was translated into NPE and integrated into EPIWATCH^®^. Out of 435 total search terms, 115 were translated into NPE by a native speaker and validated against usage in BBC Pidgin articles [19] and Nigerian social media content to ensure accuracy and contextual relevance. These NPE terms were then included into EPIWATCH^®^ keyword matrix for epidemic surveillance, which serves as the input layer for the platform’s outbreak detection pipeline. As previously described, EPIWATCH^®^ employs automated web-scraping, natural language processing (NLP), and machine learning classifiers to filter outbreak-related content, identify relevant epidemic signals, and generate early alerts [15, 16, 33, 36]. By expanding EPIWATCH^®^ search terms to include NPE terms, the same algorithmic processes that detect signals in other languages were applied to NPE content. Importantly, the integration did not modify the underlying AI models but enhanced the platform’s linguistic coverage, thus allowing surveillance of outbreak-related news and social media reports written in NPE.

In NPE, many medical words are being used specifically because they are widely accepted as such. When translating the terms, we focused on keeping the meanings clear and simplifying the language. This way, they can be easily understood in the NPE or local context. This is especially important when describing symptoms linked to specific syndromes or diseases. As the untranslated terms are widely recognized in the medical setting, they are frequently used without a literal translation. The translation phase was completed in three weeks, followed by rapid integration into EPIWATCH^®^, over two days. Prospective data collection occurred from March 5 to April 5, 2024, following the integration of NPE into EPIWATCH^®^, enabled comparative analysis with the five-year retrospective surveillance data to assess the impact of NPE on surveillance in Nigeria. Specifically, common outbreaks from March 2024 were compared with those from prior years.

Analysis of the regional distribution of diseases was done by studying disease patterns in different states in Nigeria. The spread of disease occurrence was assessed and reported outbreaks were mapped to visualize the distribution of diseases. Variables like environmental factors were taken into consideration to understand the geographical distribution of diseases.

## Results

### Descriptive Epidemiology

This analysis focuses on examining the trends of various diseases in Nigeria over the five-year period from 2018 to 2023. Overall, there were fluctuations in the burden of disease in Nigeria during the study period.

**Fig. 1:**
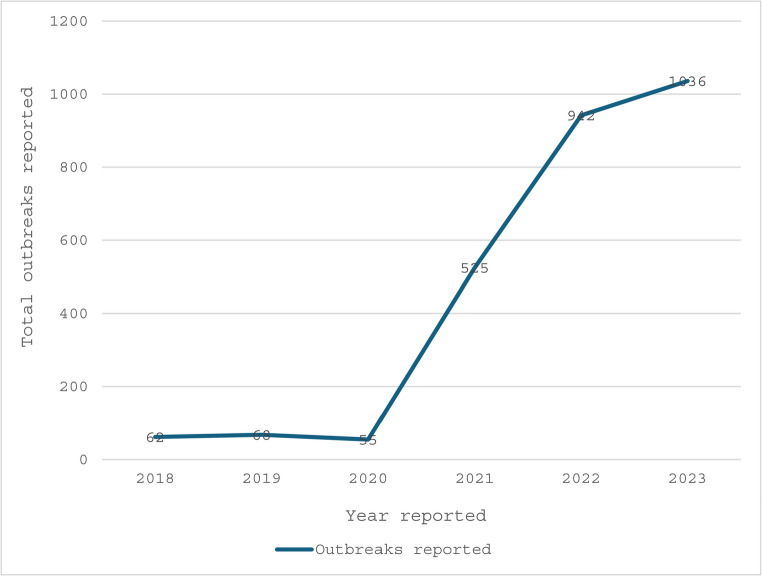
Annual trend of outbreaks reported in Nigeria between 2018 and 2023, based on EPIWATCH^®^ data

As shown in Fig. 1 below, 2,688 outbreak reports were generated between January 2018 and December 2023. With 1,036 (39%) outbreak reports, 2023 recorded the most, followed by 2022 with 942 (35%) reports. In 2020, the fewest outbreaks were reported in the last four months of the year, with only 55 (2%) outbreaks.

**Fig. 2:**
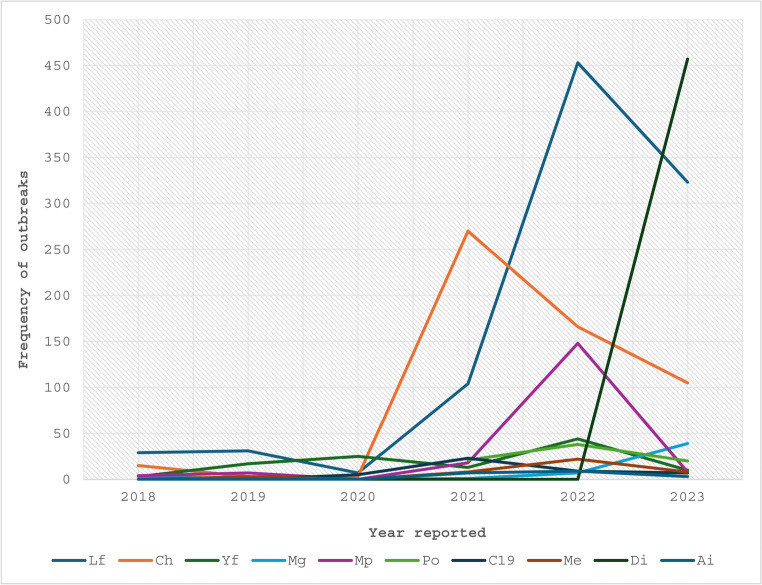
Annual trend of the top 10 reported outbreaks in Nigeria over a five-year period, based on EPIWATCH^®^ data, *Lf – Lassa fever *Ch – Cholera *Yf – Yellow fever *Mg – Meningitis *Mp – Mpox *C19 – COVID-19 *Me – Measles *Di – Diphtheria *Po – Poliomyelitis *Ai – Avian Influenza

Except for 2020, which witnessed a rise in reported yellow fever outbreaks and a decrease in reported Lassa fever outbreaks, cholera and Lassa fever were the two most common diseases in reports for years 2018, 2021, 2022, and 2023 as shown in Fig. 2. In 2022, outbreaks of Mpox accounted for 16% of all reported outbreaks, with Lassa fever carrying the largest burden at 48%, followed by cholera at 18%. Of all the diseases reported in 2023, cholera accounted for 10%, Lassa fever for 31%, and the diphtheria outbreak for 44%. Throughout the 5-year period, there were also reports of occasional poliomyelitis and meningitis outbreaks all year round.

**Fig. 3:**
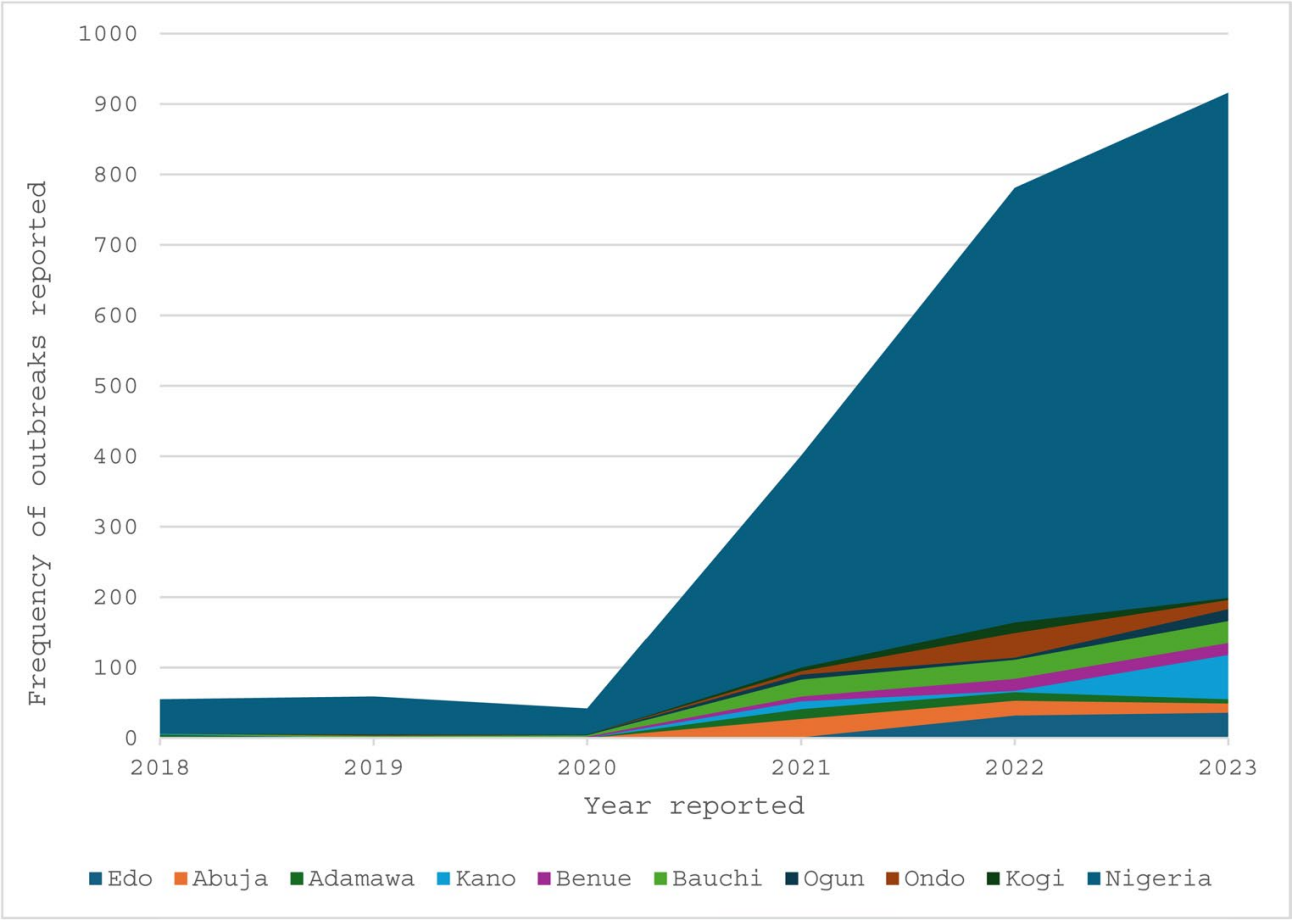
Geographical distribution of the major diseases reported in Nigeria between 2018 and 2023, derived from EPIWATCH^®^ data

Apart from Edo and Ondo, which saw a steady increase in reported Lassa fever outbreaks from 2018 to 2023, the states in the Northern region of Nigeria were disproportionately affected by more diseases than any other state in the country (Fig. 3).

## Comparative Review

To assess the impact of integrating NPE into EPIWATCH^®^, we compared the frequency of reported outbreaks in March 2024 (post-integration) with the baseline median frequency of outbreaks from March across the five preceding years (2018–2023). Because the data did not follow a normal distribution, the median was chosen to reduce the influence of outliers. As shown in the revised Table 1, the median number of total outbreaks reported between 2018 and 2023 was 27, compared to 112 outbreaks detected in March 2024 following NPE integration. This represents a 315% increase in EPIWATCH^®^ outbreak reports in March 2024 than the March median for the preceding time period, following NPE language integration.

When examined by disease, marked increases were observed for meningitis (0 vs. 10 outbreak reports), yellow fever (0 vs. 5), and diphtheria (0 vs. 12), while Lassa fever showed the most substantial increase (29 vs. 73 signals, + 152%). These findings suggest that the inclusion of NPE terms substantially enhanced the platform’s ability to detect outbreak signals in open source data.

**Table 1:** Comparison of the five top reported outbreaks in March before and after NPE integration, derived from EPIWATCH^®^ data

Disease	*Median (2018–2023)	2024 (After NPE integration)	% Change
Yellow fever	0	5	+ 5 (absolute)
Polio	0	3	+ 3 (absolute)
Meningitis	0	10	+ 10 (absolute)
Diphtheria	0	12	+ 12 (absolute)
Lassa fever	29	73	+ 152%
Total	27	112	+ 315%

*Median of frequencies from 2018 to 2023.*Percent change is not defined where the baseline value is zero; absolute differences are reported instead. *All counts presented here refer to EPIWATCH® outbreak reports derived from open source signals; they should not be interpreted as confirmed surveillance case numbers.

March outbreak data for the top five diseases across 2018–2024 are presented in Table 2, which shows how the number of March outbreaks for the five major diseases changed between 2018 and 2024. Lassa fever is the most consistently reported, while sudden rises in diphtheria and meningitis in 2023 and 2024 indicate new and emerging threats. Across the study period, the pattern indicates that the integration of NPE into EPIWATCH^®^ in 2024 enhanced the system’s ability to detect outbreak signals compared with earlier years.

**Table 2:** Annual March outbreaks of the top five diseases reported between 2018 and 2024, based on EPIWATCH^®^ data

Disease	2018	2019	2021	2022	2023	2024
Yellow fever	0	0	0	0	0	5
Poliomyelitis	0	0	0	5	4	3
Meningitis	2	0	0	0	0	10
Diphtheria	0	0	0	0	28	12
Lassa fever	5	5	29	63	37	73
**Total**	9	6	42	96	72	112

*March 2020 data have been excluded from the historical table because the data for this period was unavailable due to COVID-19 disruptions.*These figures represent EPIWATCH^®^ outbreak reports derived from open source signals; they should not be interpreted as confirmed surveillance case numbers.

## Discussion

This study assessed the impact of integrating NPE into the EPIWATCH^®^ epidemic intelligence system and to determine whether this linguistic inclusion would enhance outbreak signal detection relevant to Nigeria. Our results demonstrated a 315% increase in EPIWATCH^®^ outbreak reports in March 2024 NPE post-integration compared to the March median for the preceding period, highlighting the value of adapting AI surveillance tools to reflect the linguistic realities of diverse populations. This is particularly important in Nigeria, where the Nigerian healthcare system’s limited ability to quickly identify, diagnose, and contain outbreaks is compounded by language diversity and communication barriers.

The overall pattern of outbreaks during the 2018–2023 review period illustrates the high infectious‑disease burden in Nigeria, with the northern region disproportionately affected. This aligns with prior reports linking environmental, climatic, and infrastructural factors to disease persistence in the region [4, 23, 38]. As in earlier studies, Lassa fever and cholera were consistently prominent [6, 48], while emerging spikes in diphtheria [42] and Mpox [35, 43] show the evolving nature of outbreak threats. By comparing our findings to historical national data, we note that the integration of NPE did not just replicate known trends, in some cases, it surfaced earlier or more granular outbreak signals than traditional systems capture.

The findings also align with a growing body of evidence that local language integration in AI‑driven surveillance improves both reach and timeliness of epidemic intelligence. For example, MacIntyre et al. [33] emphasizes that multilingual data processing is crucial for effective early detection of infectious disease outbreaks. Similarly, Joshi et al. (2020) [16] found that language integration with social media data, specifically tweets, improved identification of early community‑level outbreak signals during the 2014 West African Ebola epidemic. Additionally, Silenou et al. [36] described how digital tools like SORMAS can support language integration in epidemic surveillance.

Our results contribute to this literature by providing quantitative evidence that adding NPE, a lingua franca widely understood across Nigeria, can meaningfully increase outbreak signal capture. As seen in other contexts, linguistic familiarity fosters greater public engagement with reporting channels, particularly in multilingual, resource‑limited settings where a single official language does not reflect daily communication norms. This mirrors patterns noted in culturally adapted OSINT projects elsewhere, where lowering language barriers improved both the volume and timeliness of detection.

By reframing the discussion to focus on these parallels, we emphasise that the benefit of Indigenous‑language integration is not limited to a single country or pathogen. Instead, it represents a scalable, evidence‑based strategy to strengthen epidemic preparedness globally. Future research should explore the integration of multiple Nigerian Indigenous languages, such as Hausa, Yoruba, and Igbo, into AI surveillance systems to assess further gains in sensitivity and inclusivity.

The findings in this study, demonstrate the high number of infectious diseases outbreaks in Nigeria, especially in the northern region [5, 34]. During the five-year period, some diseases displayed an increasing trend, while others showed a declining trend. These differences could be attributed to advancements in disease surveillance, raised public awareness of health issues, and the use of focused interventions [6, 10, 35–37].

Generally, outbreaks tend to peak during the dry season, correlating with increased rodent activity and food scarcity [38, 39]. As shown in Fig. 1 above, the fewest outbreaks were reported in 2020, which was probably caused by the COVID-19 era reporting gap or delayed surveillance systems that was linked to overburdened health systems in low resourced countries [40, 41]. The pattern of the trend of reported outbreaks over the five-year period was not consistent [42]. Apart from the increased reported diphtheria outbreak in 2023[43] and that of Mpox in 2022 [44], Lassa fever and cholera outbreaks were reported nearly year-round during the five-year period [45, 46].

The increase in outbreak detection following the integration of NPE into EPIWATCH^®^ matches findings from other contexts where adapting language or culture, improved surveillance capacity. For instance, involving multiple languages has been shown to increase participation and reporting in multilingual health systems [21, 25, 27, 28]; supporting our observed post-NPE integration observations. Similarly, reports from other culturally specific OSINT and syndromic surveillance projects [14, 15, 25] indicate that lowering language barriers can enhance case capture efficiency and timeliness.

The geographic concentration of outbreaks in northern Nigeria reflects patterns mentioned by Ibrahim et al. [4, 23] and Redding et al. (2021) [38] i.e. environmental, climatic, and infrastructure factors that drive high disease burden in this area. The prominence of Lassa fever and cholera in our dataset corresponds with the endemic profiles described by Dalhat et al. (2022) [6] and Elimian et al. (2020) [48]. However, our results suggest more consistent year-round reporting, likely showing that OSINT-based detection is more sensitive than traditional methods.

In contrast, our discovery of a sharp rise in reported diphtheria in 2023 stands out from the longer-term national decline noted by Abdulrasheed et al. (2023) [43]. This difference may stem from both a real outbreak resurgence and better case identification due to expanded NPE search capabilities. Likewise, although Mpox was reported sporadically in both our study and Silenou et al. (2020) [36], our spike in 2022 was proportionally larger. This could be due to increased media attention and OSINT sensitivity following global case rises.

These comparisons indicate that the NPE integration did not just mirror trends found in earlier studies; in some instances, it uncovered more detailed or earlier signals. This implies that culturally and linguistically adapted OSINT platforms can support, and in some situations, surpass traditional reporting systems in both speed and thoroughness. This improvement enhances the overall surveillance structure in resource-limited areas.

### Common Outbreaks in Nigeria during the five-year Period

- **Lassa Fever**: With outbreaks occurring frequently throughout Nigeria, Lassa fever remains a serious public health problem there. The incidence of Lassa fever outbreak reports from open sources has increased alarmingly over the past five years. Several factors contribute to the spread of the disease, such as inadequate healthcare infrastructure, poor sanitation and limited access to treatment and diagnostic services. Early detection, case management, and community-based interventions have been the mainstays of efforts to contain Lassa fever outbreaks; however, difficulties still exist in guaranteeing an efficient and prompt response to outbreaks.
- **Cholera**: Another major threat to public health in Nigeria is cholera outbreaks, which are especially common during the rainy season. Over the past five years, there has been variability in the incidence of cholera, with periodic spikes in signals reported in different states. In afflicted communities, cholera is spread by poor sanitation, tainted water sources, and overcrowding. Clean water supply, hygienic education, immunization campaigns, and better case management in medical facilities are examples of control measures.
- **Yellow Fever**: Although fewer often occurring, yellow fever outbreaks are still a concern in Nigeria. Over the previous five years, there has been fluctuation in the incidence of yellow fever, with intermittent outbreaks taking place in various areas. Initiatives to vaccinate high-risk groups have aided in slowing the disease’s spread.
- **Diphtheria**: During the previous five years, there has been a decrease in the incidence of this vaccine-preventable disease in Nigeria. Diphtheria signals have decreased in part because of routine vaccination with the diphtheria-tetanus-pertussis (DTP) vaccine. To keep the spread of diphtheria under control, regular immunization programs must be strengthened, surveillance must be improved, and suspected signals must have prompt access to medical care.
- **Mpox**: Over the past five years, there have been intermittent reports of Mpox outbreaks in Nigeria, despite the disease being comparatively uncommon in comparison to other illnesses. To stop the spread of Mpox, surveillance programs and public health initiatives like case isolation and contact tracing are crucial. Early detection and response to outbreaks can be facilitated by increasing public and healthcare provider knowledge of the signs, symptoms, and mode of transmission of Mpox.

The significant number of reported outbreaks recorded over the five-year study period poses a concern because many of those diseases have a high morbidity and death rate, especially when early diagnosis and proper treatment are lacking. These findings support earlier reports of endemicity in Nigeria and other West African countries [47]. The substantial number of outbreaks reported in Edo and Ondo could be caused by ecological and cultural practices, or by the reservoir’s existence in the states; besides having a sizable treatment facility in Edo State, where many of the country’s Lassa fever cases are handled, food is also dried outside [39, 48]. It is not recommended to rely solely on prior reports of seasonal variations in case occurrence for preparatory activities, considering the nearly year-round outbreak frequency during the five-year study period and the asymmetrical pattern of peak occurrence of confirmed cases. It also supports recorded accounts that different outbreaks can happen at any time of the year. To successfully monitor and respond to disease trends and guarantee ongoing advancements in disease control and prevention initiatives, it is essential to maintain improved surveillance measures. Our analysis illustrates the geographic distribution of reported outbreaks in all the 36 states and the geopolitical zones [39]. The geographical distribution pattern suggests that targeted public health interventions are needed in the northern region to mitigate the impact of outbreaks and improve overall health outcomes in these areas.

A review of health patterns in Nigeria over the previous five years indicates a complicated interaction between different infectious diseases. Effective immunization campaigns have led to a decline in some diseases, like diphtheria, but the public health remains seriously threatened by others, like Lassa fever and cholera [6, 49]. The persistence of these diseases is attributed to a few factors, including limited access to healthcare services, poor sanitation, and inadequate healthcare infrastructure. Furthermore, the periodic emergence of illnesses such as Mpox and yellow fever highlights the significance of strong surveillance systems and quick reaction times in managing infectious diseases in Nigeria [44, 50]. Comprehensive strategies that combine efforts in prevention, detection, and response are necessary to effectively address the burden of these diseases [4, 5]. This entails bolstering vaccination rates, boosting access to necessary services, encouraging good sanitation and hygiene habits, and fortifying healthcare systems. To reduce the burden of infectious diseases in Nigeria and achieve long-lasting improvements in public health outcomes, cooperation between government organizations, healthcare providers, and foreign partners is essential. Also, enhanced surveillance and early detection are essential to this collaboration because they allow for the prompt identification and containment of disease outbreaks, preventing the spread of infectious diseases and lessening their effects on the populace.

A major step toward enhancing disease surveillance in Nigeria is the integration of NPE into EPIWATCH^®^. This approach emphasizes inclusive strategies to effectively address health challenges, while acknowledging the significance of cultural and linguistic diversity in public health interventions. By comparing the common outbreaks from March 2024 with those from previous years using EPIWATCH^®^, one can gain insight into how Nigeria’s disease patterns are evolving. Since certain diseases may show consistent trends over time while others may show fluctuations or emerge as new challenges, an understanding of these temporal variations is crucial for customizing public health interventions to address evolving health needs and priorities. The results hold great significance for public health response tactics in Nigeria, as improved surveillance capacities allow officials to identify epidemics sooner, take immediate action, and deploy resources more effectively. This proactive strategy is essential for preventing the spread of disease, reducing morbidity and mortality, and eventually enhancing population health outcomes. The integration of NPE into EPIWATCH^®^, demonstrates the potential of using OSINT to improve disease surveillance and response.

While BBC News archives could, in theory, be used to retrospectively apply NPE search terms over the 2018–2023 period for a head to head comparison of detection rates, the limited prevalence of NPE in that source and its lack of extensive translation data reduce its suitability as a standalone comparator. However, the concept of a retrospective, same-period analysis remains compelling. A future study could implement this approach across a broader range of historical open source media and social platforms where NPE is more widely used, enabling a truer comparable evaluation of outbreak detection with and without NPE integration.

## Limitations

This study has several limitations. First, while EPIWATCH draws on a broad range of OSINT inputs, there is inherent variability in the quality, completeness, and timeliness of online reports. Signals may be missed due to reporting lags, inconsistent terminology, or the absence of coverage in the sources monitored. Second, although the integration of NPE substantially broadened linguistic reach, other widely spoken indigenous Nigerian languages such as Hausa, Yoruba, and Igbo were not included in this analysis. Their absence may under-represent outbreak signals from populations where those languages predominate.

While a retrospective comparison using NPE search terms on archived BBC News content from 2018 to 2023 could have given another baseline for detecting outbreaks without NPE integration, this was not done because relevant NPE material is limited in the BBC News archives [19]. BBC reporting on Nigerian events is mainly in standard English, with few colloquial or indigenous language pieces. The archive does not provide a large database for translation. Relying on this single source would create sampling bias and likely underestimate NPE specific signals. Future studies could use this comparative approach with a wider range of historical news and social media sources, including Nigerian online sites, regional broadcast transcripts, and community reporting platforms, where NPE content is more common.

Comparing outbreaks before and after integrating NPE into EPIWATCH^®^ may not fully reflect the broader implications for disease surveillance and response due to limitations such as time frame i.e., one-month prospective review, scope of analysis, contextual factors, etc. and methodological challenges. These drawbacks imply that the impact of NPE integration on disease surveillance and response might be more intricate and varied than a straightforward before-and-after analysis could adequately convey [43–45]. While not specifically examined in this comparison, other aspects of disease surveillance and response, such as community engagement, intervention efficacy, and overall public health outcomes, may also affect the diseases trends seen in this study. Additional limitations may include the applicability of the results outside Nigeria.

Finally, as with other OSINT based approaches, results reflect what is publicly available and may not capture unreported outbreaks or those detected solely through formal surveillance channels. These factors should be considered when interpreting the findings.

## Conclusion

Linguistic and cultural diversity [21, 27, 28] is crucial for public health interventions, enabling inclusive strategies that strengthen outbreak response [33]. This study has shed light on how Nigerian disease patterns are changing by comparing outbreaks from March 2024 with those from prior years and emphasizes the need for better surveillance to enable early detection and more effective resource allocation, and to understand temporal variations for customized public health interventions. OSINT is a valuable resource which can improve surveillance and response efforts, as shown by the effective integration of NPE into EPIWATCH^®^.

This study’s strength lies in its innovative integration of NPE into an AI-driven surveillance system, addressing a major communication barrier in outbreak detection. By leveraging a large five-year dataset, the analysis demonstrated a measurable 315% increase in EPIWATCH^®^ outbreak reports after language integration. These findings highlight the value of combining linguistic inclusivity, AI, and OSINT to strengthen disease surveillance in multilingual, resource-limited settings. They also demonstrate the importance of NPE integration and technological advancements in enhancing Nigeria’s capacity for early detection and response. Compared with traditional approaches, open-source early warning systems offer substantial advantages. EPIWATCH^®^ provides real-time surveillance of diseases and syndromes, enabling timely formal investigations prompted by emerging signals.

## Supplementary Information

Below is the link to the electronic supplementary material.

Supplementary Material 1

## Data Availability

All data underlying the findings are available on reasonable request. To support reproducibility, the Nigerian Pidgin keyword set used in the analysis has been provided as Supplementary File 1.

## Declarations

This study is based on a comprehensive data set on infectious diseases in Nigeria over a five-year period. The data set contains information about the types of infectious diseases reported, the number of signals, the locations and dates of the outbreaks reported, and possibly additional demographic or clinical details. In addition, data is available on the integration of NPE into EPIWATCH^®^, which includes the development and implementation of the language integration as well as any relevant results or impact assessments.
